# Preparation and Characterization Study of Zein–Sodium Caseinate Nanoparticle Delivery Systems Loaded with Allicin

**DOI:** 10.3390/foods13193111

**Published:** 2024-09-28

**Authors:** Ling Hu, Pengcheng Zhao, Yabo Wei, Yongdong Lei, Xin Guo, Xiaorong Deng, Jian Zhang

**Affiliations:** 1 School of Food Science and Technology, Shihezi University, Shihezi 832003, China; 2 Key Laboratory of Characteristics Agricultural Product Processing and Quality Control (Co-Construction by Ministry and Province), Ministry of Agriculture and Rural Affairs, School of Food Science and Technology, Shihezi University, Shihezi 832003, China; 3 Key Laboratory for Food Nutrition and Safety Control of Xinjiang Production and Construction Corps, School of Food Science and Technology, Shihezi University, Shihezi 832003, China

**Keywords:** allicin, zein, sodium caseinate, nanoparticle

## Abstract

Allicin, as a natural antibacterial active substance from plants, has great medical and health care value. However, due to its poor stability, its application in the field of food and medicine is limited. So, in this paper, allicin–zein–sodium caseinate composite nanoparticles (zein–Ali–SC) were prepared by antisolvent precipitation and electrostatic deposition. Through the analysis of the particle size, ζ-potential, encapsulation efficiency (EE), loading rate (LC) and microstructure, the optimum preparation conditions for composite nanoparticles were obtained. The mechanism of its formation was studied by fluorescence spectrum, Fourier infrared spectrum (FTIR), X-ray diffraction (XRD) and thermogravimetric analysis (TGA). The stability study results showed that the particle size of composite nanoparticles was less than 200 nm and its PDI was less than 0.3 under different NaCl concentrations and heating conditions, showing good stability. When stored at 4 °C for 21 days, the retention rate of allicin reached 61.67%, which was 52.9% higher than that of free allicin. After freeze-drying and reheating, the nanoparticles showed good redispersibility; meanwhile, antioxidant experiments showed that, compared with free allicin, the nanoparticles had stronger scavenging ability of free radicals, which provided a new idea for improving the stability technology and bioavailability of bioactive compounds.

## 1. Introduction

As the main bioactive substance extracted from garlic, allicin (diallyl thiosulfinate) has long been valued and discovered to have various utilization values [1]. Relevant studies have confirmed that allicin is equally effective as conventional antibiotics such as beta-lactam or glycoside antibiotics, can effectively inhibit the growth of *Staphylococcus* aureus, and has a good scavenging effect on 1,1-diphenyl-2-picrylhydrazyl (DPPH) and other free radicals [2]. Allicin can also reduce the risk of different types of tumors such as lung cancer, stomach cancer, and colon cancer [3]. Therefore, allicin has broad application prospects as a dietary supplement or a drug for the prevention and treatment of diseases. However, because allicin is hydrophobic, volatile, and extremely sensitive to surrounding conditions, its application in food and medicine is limited. Therefore, more and more research is devoted to improving the water solubility and chemical stability of allicin.

With the development of nanotechnology in food, medicine and other fields, the potential of nanoscale carrier systems to encapsulate and deliver hydrophobic bioactive substances to improve the water dispersion, chemical stability, release properties and biological efficacy of compounds has been increasingly recognized by researchers [4]. Drug delivery systems based on nanoparticle carriers have been applied in the treatment of rheumatoid arthritis, tumor and other diseases, while composite nanoparticle carriers of bioactive substances are also developing rapidly in the fields of fruit and vegetable preservation and functional food development. Existing studies usually use animal and plant proteins or polysaccharides to construct food-grade delivery systems. Among them, zein has a relatively ideal effect on improving the bioavailability of active substances due to its wide source, low price, and the advantages of amphiphilicity and good biocompatibility [5,6]. For example, Yao et al. [7] showed that when curcumin was coated with zein nanoparticles, its in vitro bio-accessibility (22.4%) was significantly higher than that without coating (<8%), and the stability of the active substance was enhanced. The inhibitory effect of thymol loaded zein nanoparticles prepared by Zhang et al. [8] on Gram-positive bacteria lasted longer than that of thymol alone. Liu et al. [9] prepared curcumin–zein nanoparticles, which not only showed higher DPPH radical scavenging activity than vitamin C on the basis of improved stability, but also showed a controlled release effect in in vitro digestion simulation. In recent years, zein has been widely used to construct nanoparticle systems as excipients for drugs, scaffolds for tissue engineering, and delivery systems for active substances [10].

Despite the obvious advantages of zein nano-delivery systems, nanoparticles composed of a single zein nanoparticle have insufficient stability in colloidal systems under high temperature, weak acidity, neutral and salt-containing ion environments, and are prone to particle aggregation and precipitation. In addition, when passing through the digestive system, some proteases in the digestive fluid will destroy the outer structure of the nanoparticles, thus failing to protect the coated bioactive compounds. Therefore, the use of hydrophilic materials (polysaccharides, proteins, synthetic polymers and surfactants) to modify, coat and stabilize zein nanoparticles to fully exploit their advantages has become a research focus. Among them, sodium caseinate can be coated with different polar compounds at the same time, and it is easy to obtain and has good physicochemical stability, making it an ideal encapsulation material. According to reports, sodium caseinate-stabilized zein nanoparticles have been successfully used to encapsulate rutin, curcumin, fucoxanthin and other active compounds [10,11,12]. The thymol–zein–sodium caseinate nanoparticles prepared by Li et al. [13] maintained the antibacterial activity and DPPH radical scavenging activity of thymol. Chang et al. [14] found that pectin coating not only significantly improved the coating ability of protein nanoparticles towards curcumin, but also had a slow-release effect under gastrointestinal conditions, showing excellent stability. Patel [15] and Alqahtani [16] et al. reported that when using sodium caseinate to stabilize zein self-assembled nanoparticles, the colloidal particles produced had excellent redispersibility and salt tolerance.

Therefore, in this study, allicin was used as the active substance, and the binding sites and force types of allicin and zein were predicted by a molecular docking method. Allicin zein nanoparticles coated with sodium caseinate were prepared by antisolvent precipitation and electrostatic deposition, and their structures were characterized to obtain composite nanoparticles with good properties. The formation mechanism of nanoparticles was analyzed, and their stability and oxidation resistance under different conditions were evaluated. In addition, the research group selected gelatin as the film-forming substrate in the early stage, prepared the allicin-loaded zein sodium caseinate composite nanoparticle gelatin film, and applied it to the storage of beef, effectively alleviating the quality deterioration of beef [17]. The results of this research not only contribute to the development of new allicin-loaded delivery systems, but also provide theoretical support for the application of allicin-loaded nano-delivery systems in food, medicine and other fields.

## 2. Materials and Methods

### 2.1. Materials

Zein (food-grade) was obtained from Shanghai Ruixiang, allicin (purity ≥ 95%) and sodium caseinate (food-grade) was obtained from Shanghai Maclin. Other regents used in this experiment were analytical-grade.

### 2.2. Molecular Docking

The molecular structure of allicin was obtained by PubChem retrieval. Using AlphaFold3 (Google) to predict protein structure, Auto-Dock-Tools was used to minimize energy and optimize the geometric structure of proteins. AutoDock Vina (Olson, AutoDock4.2) was used to perform global docking, and the docking results were analyzed using Protein-Ligand Interaction Profiler (PLIP). The docking results were visualized using PyMOL (Schrödinger, 3.01). After the conversion of the pdbqt format, the interaction mode and binding ability between allicin and zein were analyzed.

### 2.3. Sample Preparation

An amount of 1.0 g of zein was dissolved in 50 mL of ethanol–water solution (80%), followed by magnetic stirring (HH-S2, Jinyi, Shanghai, China) for 1 h (600 r/min); then, 0.10 g allicin was added, stirring was continued for 1 h, and the mixture was centrifuged (Mulltifuge X1R, Thermo Field, Waltham, MA, USA) for 15 min (5000 r/min) to remove insoluble impurities. The resulting sample was stored at 4 °C for future use.

Sodium caseinate was weighed and dissolved in 100 mL pure water, stirred magnetically at 800 r/min for 3 h, and centrifuged for 15 min (5000 r/min). The supernatant as sodium caseinate was taken as a reserve solution and stored at 4 °C for future use.

An amount of 10 mL of the prepared allicin zein solution was measured and added to 40 mL sodium caseinate solution. Magnetic stirring was conducted for 3 min (600 r/min) to obtain zein–Ali–SC dispersions. The ethanol was removed by rotary evaporation (RE-52, Yarong, Zhongshan, China) (40 °C, −0.1 MPa) and made to a volume of 50 mL with pure water. Stirring was continued for 5 min (600 r/min), followed by centrifugation at 5000 r/min for 15 min, and the supernatant was taken, which was a zein–Ali–SC sample.

### 2.4. Particle Size, Polydispersity Index (PDI) and ζ-Potential

After diluting the nanoparticle dispersion by ten times (to reduce multiple scattering effects), the particle size, PDI, and ζ-potential were analyzed using a laser particle size analyzer (Nano-ZS90, Malvern Panalytical, Worcestershire, UK).

### 2.5. EE and LC

An amount of 1 mL of nanoparticle dispersion was sonicated (KQ-200VDE, Kunshan Ultrasonic Instrument, Kunshan, China) for 1 minute, to which 80% ethanol aqueous solution (*v*/*v* = 1:100) was added. Magnetic stirring was conducted for 30 min, followed by centrifugation for 15 min (8000 r/min), and then allicin content was determined with the supernatant solution. Absorbance was measured (412 nm) with a UV-VIS spectrophotometer (T3200, Youke, Beijing, China). EE and LC were calculated as shown below:

(1) $$\mathrm{EE}\;(\%)=\frac{M_{0}}{M_{1}}\times 100$$

(2) $$\mathrm{LC}\;(\%)=\frac{M_{0}}{M_{2}}\times 100$$

Here, M_0_ represents the quality of allicin in nanoparticles, M_1_ represents the quality of allicin, and M_2_ represents the sum of the quality of allicin, zein, and sodium caseinate.

### 2.6. Microscopic Morphology Observation

The nanoparticles were freeze-dried (FD-A10N-50, KuanSon, Shanghai, China) and sprayed with gold under vacuum for 120 s. The microstructure of the nanoparticles was observed by scanning electron microscopy (SEM) (SU8010, Hitachi, Tokyo, Japan).

### 2.7. Structural Characterization

#### 2.7.1. Fluorescence Spectrum

The nanoparticle dispersion was diluted (zein is 0.25 mg/mL) and measured using a fluorescence spectrometer (Card-F98, Precision Instruments, Shanghai, China). Both the excitation and emission slit width were 5 nm, the scanning wavelength was 290–450 nm, and the excitation wavelength was 280 nm.

#### 2.7.2. FTIR

The freeze-dried samples were mixed with KBr and then laminated, followed by scanning 16 times with a resolution of 4 cm^−1^ using an FTIR (Bruker Vertex 70v Prior, Karlsruhe, Germany) in the wavelength range of 4000–400 cm^−1^.

#### 2.7.3. XRD

The crystal morphologies of zein–Ali–SC, zein–SC, zein and sodium caseinate were determined by XRD (X’Pert-Pro MRD, Panalytical, Beijing) with a fixed 2θ scanning range of 5–90° and a speed of 5°/min.

#### 2.7.4. TGA

An amount of 10 mg of the sample was weighed and, TGA was performed using a thermogravimetric analyzer (STA 449F5, Netzsch, Hanau, German). The temperature range was set to 30–600 °C and the heating rate to 20 °C/min.

### 2.8. Stability under Different Conditions

#### 2.8.1. pH

The freshly prepared zein–Ali–SC dispersion was adjusted to different pH (3, 4, 5, 6, 7, 8) with HCl (0.1 mol/L) and NaOH (0.1 mol/L), and the particle size and PDI were determined after standing at 4 °C for 24 h.

#### 2.8.2. NaCl Concentration

Different amounts of NaCl were added to the freshly prepared nanoparticle dispersion (0, 0.1, 0.2, 0.25, 0.5 mol). This was stirred and mixed evenly with magnetic stirring and left to stand for 2 h. The particle size and ζ-potential were measured.

#### 2.8.3. Thermal Stability

An amount of 10 mL of freshly prepared zein–Ali–SC dispersion was heated in an 80 °C water bath for different time periods (20, 40, 60, 80, 100, 120 min), and then cooled to 25 °C and left for 2 h. The particle size and ζ-potential changes of the nanoparticles were measured before and after heating.

#### 2.8.4. Storage Stability

The prepared zein–Ali–SC dispersion was stored at 4 °C (away from light) for 21 days, and the allicin content was determined every 3 days to evaluate the storage stability.

#### 2.8.5. Redispersibility

An amount of 5 mg of zein–Ali–SC was weighed after freeze-drying and added into 25 mL pure water. This was stirred magnetically for 30 min (600 r/min) to fully dissolve it. The particle size of the nanoparticles was measured before and after freeze-drying, as well as those dissolved again in water.

### 2.9. Antioxidant Activity

#### 2.9.1. DPPH Scavenging Activity

We referred to Liu’s [18] method and modified it slightly. An amount of 1 mL of zein–Ali dispersion, zein–Ali–SC dispersion, allicin solution and 1 mL DPPH solution (200 mM, dissolved in anhydrous ethanol) were mixed and incubated at 25 °C away from light for 30 min. An amount of 1 mL of anhydrous ethanol was mixed with 1 mL DPPH solution as a blank to determine absorbance (517 nm). The calculation formula is shown in (3):

(3) $$\mathrm{DPPH}\;\mathrm{radical}\;\mathrm{scavenging}\;\mathrm{activity}\;(\%)=\frac{A_{c}-A_{0}}{A_{0}}\times 100$$

Here, A_C_ represents the absorbance value of the control group, and A_0_ represents the absorbance value of the experimental group.

#### 2.9.2. 2,2′-Azinobis-(3-ethylbenzthiazoline-6-sulphonic acid) Diammonium Salt (ABTS) Radical Scavenging Activity

We referred to Meng’s [19] method and modified it slightly: 50 μL zein–Ali dispersion, zein–Ali–SC dispersion, allicin solution and 1 mL ABTS working liquid were mixed, and 1 mL ABTS working liquid was mixed with 50 μL anhydrous ethanol as the control. These were incubated at 25 °C in a dark environment for 30 minutes. The absorbance value was measured at 734 nm, and the calculation formula is shown in (4):

(4) $$\mathrm{ABTS}\;\mathrm{radical}\;\mathrm{scavenging}\;\mathrm{activity}\;(\%)=\frac{A_{c}-A_{0}}{A_{0}}\times 100$$

### 2.10. Data Statistics and Analysis

SPSS 25.0 statistical software (IBM SPSS Inc., Chicago, IL, USA) was used to perform one-way ANOVA on the data, and the obtained data results were represented by mean value and standard deviation; Origin 2020 software (OriginLab, MA, USA) was used for plotting.

## 3. Results and Discussion

### 3.1. Molecular Docking Analysis

**Figure 1:**
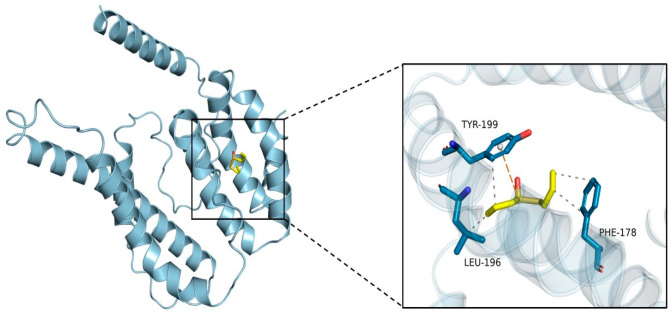
Molecular docking of allicin and zein.

Molecular docking technology is widely used to study the binding situation between two or more molecules and analyze their interaction mechanism, involving spatial- and energy-matching between molecules. The molecular docking method has a wide range of applications in drug design, material design, the exploration of natural compounds’ synthesis pathways and other fields. Therefore, the molecular docking method was used to verify the interaction and binding mode of zein and allicin. The docking results are shown in Figure 1. The 3D model of zein presented a V-shaped structure, and the binding energy with allicin was −3.7 kcal/mol. The figure shows that allicin molecules were embedded in the cavity of zein and interacted with amino acid residues. Binding sites mainly included PHE178, LEU196, and TYR199; the distances were 3.74 Å, 3.69 Å and 3.69 Å, respectively. These amino acid residues formed a hydrophobic pocket enclosing allicin molecules, which indicates that there was a hydrophobic interaction between zein and allicin, which had important significance for stabilizing allicin in zein molecules. In addition, TYR199 forms π–cation interactions with allicin. The above structural prediction, model evaluation and binding site prediction provide structural support information for the interaction between zein and allicin.

### 3.2. Particle Size and ζ-Potential Analysis

**Figure 2:**
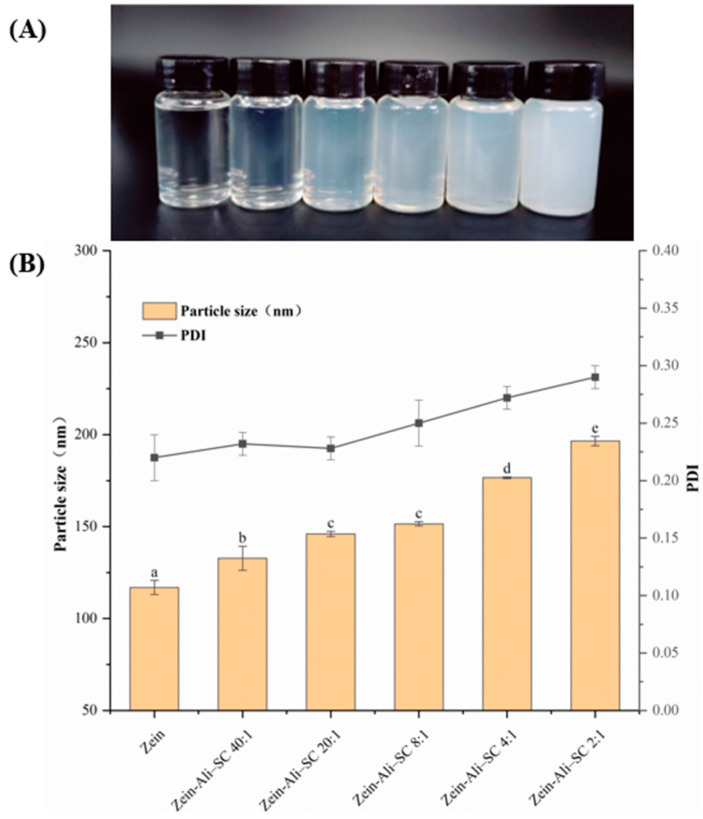
Changes in appearance (**A**), particle size, and PDI (**B**) of composite nanoparticles under different amounts of sodium caseinate addition. different lowercase letters denote significant differences (*p* < 0.05).

In this study, the effects of different sodium caseinate additions on nanoparticles were comprehensively evaluated based on particle size, ζ-potential, EE and LC. As shown in Figure 2B, with the increase in sodium caseinate addition, the particle size and PDI show a significant increasing trend. Figure 2A also reflects that the turbidity of the nanoparticle dispersion showed an increasing trend. When W_zein_/W_SC_ is 2:1, the particle size reaches its maximum 196.5 nm and the PDI is 0.291. Although there is no obvious particle aggregation phenomenon, the excess sodium caseinate molecules in the outer layer crosslink with each other, and the dispersion solution presents turbidity. A similar phenomenon was reported by Chen et al. [20]. This may be due to the electrostatic forces acting between molecules. This conclusion can also be confirmed by the ζ-potential results. Generally, ζ-potential can reflect the stability of nanoparticle dispersion system to a certain extent [21]. The larger the absolute value of the ζ-potential, the less easily the particles precipitate and aggregate because of electrostatic repulsion, thus forming a relatively stable colloidal dispersion solution. When the mass ratio of zein and sodium caseinate changes, the charge ratio of zein and sodium caseinate will change, which directly affects the interaction force between zein and sodium caseinate, and thus affects the stable state of the nanoparticles formed. Zein has a positive charge of 29.34 mV, and the zein–Ali–SC formed by adding sodium caseinate presents a negative charge. When W_zein_/W_SC_ is 8:1, ζ-potential reaches its maximum of −35.53 mV, indicating that sodium caseinate at this concentration can completely coat the surface of the nanoparticles and reach saturation. The outer charge characteristics of the composite nanoparticles are dominated by sodium caseinate, and the particles remain stable due to the electrostatic repulsion of negative charge and the steric hindrance formed by sodium caseinate, forming dense and stable core–shell-structured nanoparticles.

### 3.3. Analysis of EE and LC

According to Table 1, with the increase in sodium caseinate addition, the EE of allicin increased from 71.54% to 86.62% (the maximum) and then decreased to 81.38%. Similarly, the LC increased from 5.56% to 7.86% and then decreased to 6.84%, which may be due to the fact that sodium caseinate has more hydrophilic groups coated on the surface of the nanoparticles, forming a dense coating structure conducive to the improvement in allicin EE and LC. Similar results were shown in the study of Sun et al. [22]: when shellac was added, the EE of curcumin in nanoparticles increased. However, when the addition of sodium caseinate was further increased, bridging between nanoparticles occurred, and allicin could not enter the hydrophobic part of zein, resulting in a decrease in EE and LC. When W_zein_/W_SC_ was 8:1, the EE and LC of allicin reached maximum values, which were 86.62% and 7.86%, respectively. The particle size was 151.5 nm, and the ζ-potential also reached its maximum absolute value of −35.53 mV. Under these conditions, the nanoparticle dispersion solution was the most stable. Based on comprehensive evaluation of EE, LC, particle size and ζ-potential, nanoparticles with a W_zein_/W_SC_ = 8:1 were selected for further research.

### 3.4. Microscopic Morphology Observation

**Figure 3:**
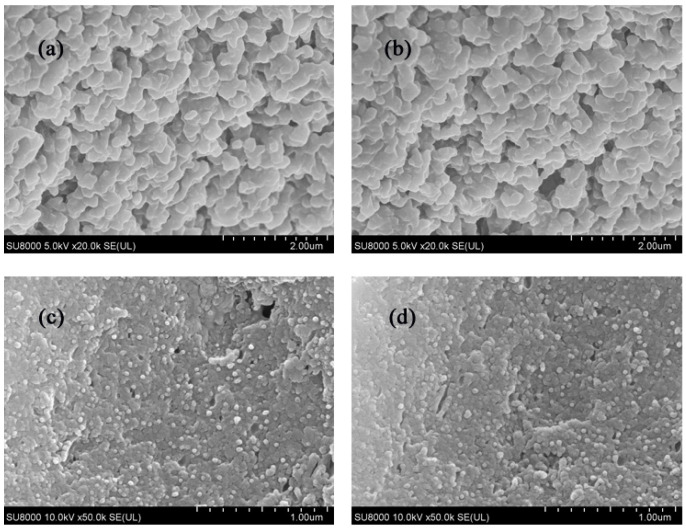
Microscopic morphology of nanoparticles: (**a**) zein, (**b**) zein–Ali, (**c**) zein–SC, (**d**) zein–Ali–SC.

Figure 3a,b show the microscopic morphology of zein and allicin–zein nanoparticles, respectively. It can be seen in the figures that both have a certain degree of aggregation phenomenon, and the connection between the nanoparticles is relatively close, which is similar to the situation reported in previous studies [23] that zein nanoparticles are highly hydrophobic and prone to aggregation. Figure 3c,d show the microscopic morphology of zein–SC and zein–Ali–SC, respectively. It can be seen in the figures that after the nanoparticles are coated with sodium caseinate, the distribution is relatively uniform. After adding allicin, zein–Ali–SC and zein–SC showed similar morphologies. These results show that the addition of sodium caseinate and allicin had no significant effect on the micromorphology of the composite nanoparticles.

### 3.5. Structural Characterization

#### 3.5.1. Fluorescence Spectral Analysis

**Figure 4:**
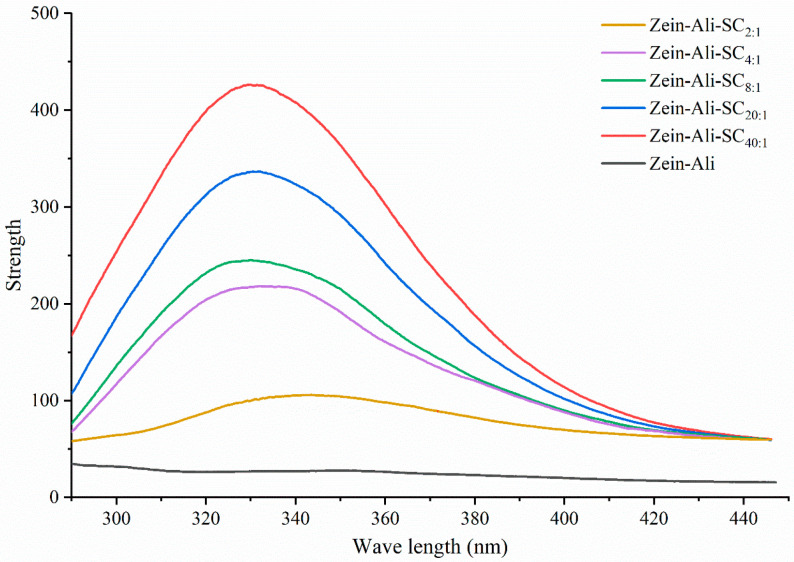
Fluorescence spectrum analysis of zein–Ali–SC with different sodium caseinate addition levels.

The presence of a high proportion of tyrosine residues in protein solution will produce endogenous fluorescence, which is extremely sensitive to the local environmental changes of the protein, so the fluorescence characteristics of tyrosine are usually selected to indicate the conformational changes of the protein in the experiment. Sodium caseinate usually has a fluorescence emission peak at 340 nm and has a very high fluorescence intensity. The effects of different sodium caseinate addition levels on the fluorescence characteristics of nanoparticles are shown in Figure 4. The position of the emission peak does not move significantly with the increase in sodium caseinate, but the fluorescence intensity shows a significant downward trend. This may be because sodium caseinate forms a stable hydrophilic coating on the outer layer of nanoparticles through electrostatic interaction and other forces, which reduces the hydrophobic amino acid residues of the nanoparticles and changes the local microenvironment, and the two undergo interactions during the polarity transition of the microenvironment, resulting in a downward trend of fluorescence intensity. Similar results were found in the study of Chen et al. [20]. With the increase in hyaluronic acid, the hydrophobic amino acids of zein–hyaluronic acid nanoparticles were shielded, the nanoparticles aggregated and the fluorescence intensity decreased.

#### 3.5.2. FTIR Analysis

**Figure 5:**
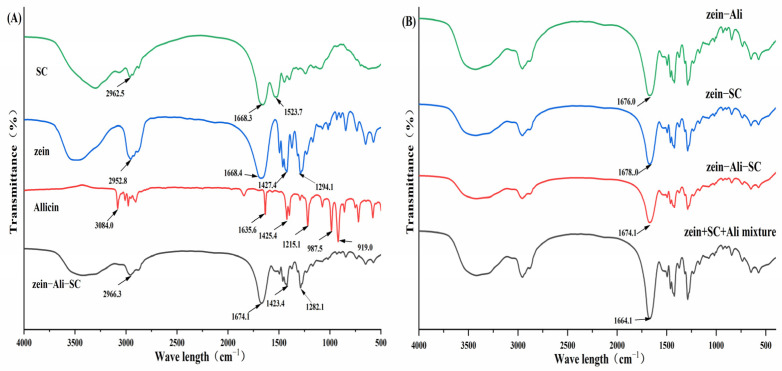
FTIR spectra of different components and nanoparticles.

As shown in Figure 5, the characteristic infrared spectra of zein mainly include the following: 2952.8 cm^−1^ (O-H stretching), 1668.6 cm^−1^ (amide I band, C=O stretching), 1538.6 cm^−1^ (amide II band, N-H bending vibration and C-N stretching vibration), and 1427.4 cm^−1^ (amide III band, C-H bending vibration) [24]. Sodium caseinate at 2962.5 cm^−1^ is mainly due to the superposition of O-H tensile vibration and N-H tensile vibration; at the same time, due to the stretching vibration of C=O and the tensile vibration of C-N, sodium caseinate exhibits characteristic peaks at 1668.4 cm^−1^ (amide I band) and 1523.7 cm^−1^ (amide II band). The characteristic peaks at the amide I band (1668.4 cm^−1^) and amide II band (1523.7 cm^−1^) are caused by C=O stretching vibration and C-N tensile vibration, respectively. Allicin has characteristic absorption peaks at 1635.6 cm^−1^ (amide I band, C=O stretching), 1425.4 cm^−1^, 1215.1 cm^−1^ and 987.5 cm^−1^.

Compared with zein, the absorption peaks of amide I band in zein–SC, zein–Ali and zein–Ali–SC moved to 1678.0, 1676.0 and 1674.1 cm^−1^, respectively, indicating the existence of electrostatic interaction during the formation of these composite nanoparticles. Chen et al. also produced similar findings [20], wherein the peaks of amide I and amide II shifted, indicating that the formation of zein–sodium caseinate composite nanoparticles involves electro-static interactions, which contribute to the formation of composite nanoparticles. However, the characteristic absorption peak of allicin could not be observed in the mixture of allicin, sodium caseinate and zein, which may be due to the degradation of allicin in the process of potassium bromide tablet, resulting in less content. In the FTIR spectra of zein–Ali and zein–Ali–SC, the characteristic absorption peaks of allicin almost disappeared or overlapped with the absorption peaks of nanoparticle materials, which may be because the encapsulation of nanocarriers limited the extension of chemical groups on allicin, which is also strong evidence that allicin successfully encapsulated into the hydrophobic interior of nanoparticles.

#### 3.5.3. XRD Analysis

**Figure 6:**
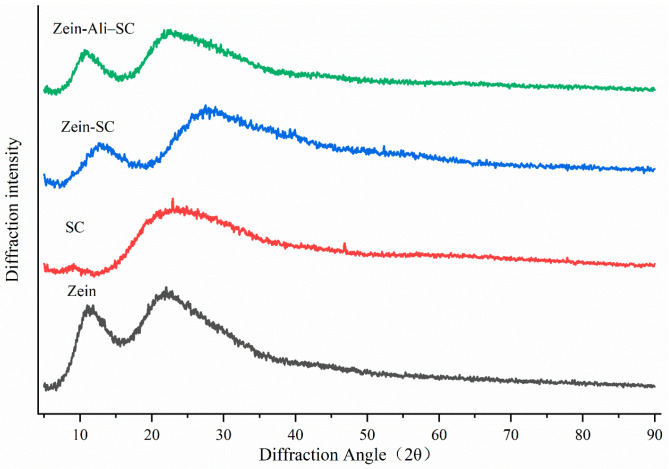
XRD analysis diagram.

The crystal state and crystal diffraction information of nanoparticles can be investigated through XRD patterns. In Figure 6, the diffraction angles 2θ of single zein and sodium caseinate are 10.3 and 20.3, 9.8 and 21.4, respectively, showing two relatively flat wide peaks without sharp characteristic peaks, which is a typical amorphous XRD pattern. The diffraction angles of zein–Ali–SC and zein–SC prepared in this study are similar, which indicates that allicin may be encapsulated in zein sodium caseinate nanoparticles in an amorphous form through non-covalent action. Wei et al. made similar findings [25]: differential scanning calorimetry and XRD patterns of resveratrol-loaded zein composite nanoparticles indicated that resveratrol had encapsulated amorphous properties.

#### 3.5.4. TGA

**Figure 7:**
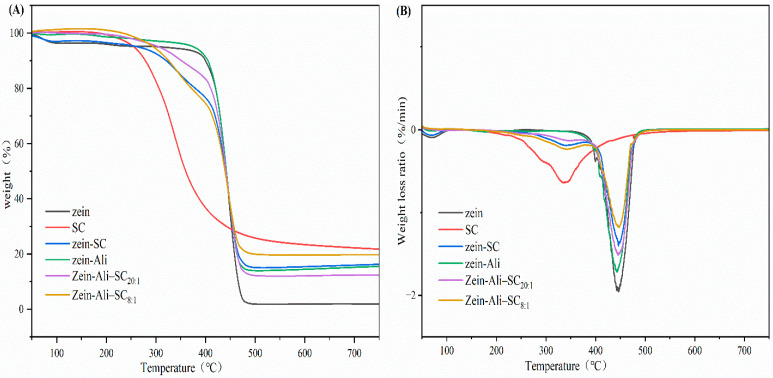
(**A**) TGA curve diagram; (**B**) DTG curve diagram.

As can be seen in Figure 7A, the TGA curve has three different temperature ranges: the first part is 50 °C to 250 °C; the second part is 250 °C to 500 °C; the third part is above 500 °C. The main cause of mass loss in the first part is the elimination of free water and bound water. The thermal degradation of sodium caseinate occurred at about 250 °C, and the degradation of zein began at about 320 °C. After adding allicin, the mass loss of zein–Ali–SC nanoparticles after thermal decomposition was lower than that of zein–SC and zein–Ali nanoparticles, and the non-covalent interaction and hydrogen bond may play a role during the formation of nanoparticles. In Figure 7B, the weight loss rate of sodium caseinate reaches its maximum at 334 °C, while that of zein reaches its maximum at 446 °C. Compared with zein, the weight loss rates of zein–SC, zein–Ali and zein–Ali–SC formed have an obvious downward trend. Zein–Ali–SC has better thermal degradation characteristics than other nanoparticles.

### 3.6. Formation Mechanism of Nanoparticles

In this study, zein–Ali–SC was prepared by antisolvent precipitation and electrostatic deposition. During the process of adding zein and allicin to the ethanol aqueous solution, the diffusion of ethanol in water changed the polarity of the system. The α-helix in zein turned into β-fold, and β-fold accumulated under the hydrophobic interaction to form a pile of nanoribbons that were deposited layer by layer to form nanoparticles; two or three zein subunits contracted to form spherical structures and aggregated to form a stable system [26,27,28]. At the same time, the hydrophobic compound allicin entered the hydrophobic part of zein under the drive of various forces such as electrostatic force and hydrogen bond, and the biopolymer self-assembled on the outer layer of zein to form composite nanoparticles through electrostatic deposition. The water dispersion system of nanoparticles was obtained by removing ethanol from the system by rotary evaporation.

The results of measuring the particle size and ζ-potential show that the formation process of the composite nanoparticles is related to the addition amount of sodium caseinate. When the amount of sodium caseinate added was relatively low (W_zein_/W_SC_ ratio of 40:1 and 20:1), the content of sodium caseinate molecule was less, which could not completely coat the surface of zein nanoparticles to form a stable hydrophilic layer, resulting in a low ζ-potential of composite nanoparticles and a low electrostatic repulsion between particles. However, when the content of sodium caseinate was high (W_zein_/W_SC_ of 8:1), the surface of zein nanoparticles was almost completely coated with negatively charged sodium caseinate molecules, and with the increase in sodium caseinate concentration, the interaction between zein nanoparticles and sodium caseinate-coated nanoparticles gradually increased. Driven by the interaction of static electricity and steric hindrance, core–shell nanoparticles with compact and stable structure were formed. But, with the further increase in sodium caseinate content (W_zein_/W_SC_ of 4:1 and 2:1), excessive sodium caseinate led to cross-linking between composite nanoparticles, increasing the particle size of the dispersion solution, and causing the nanoparticles to tend to aggregate with each other, resulting in a gradually cloudy appearance of the dispersion solution. These formation mechanisms were also strongly confirmed by the physical stability results of the composite nanoparticles.

### 3.7. Stability Analysis

#### 3.7.1. Stability Analysis at Different pH

**Figure 8:**
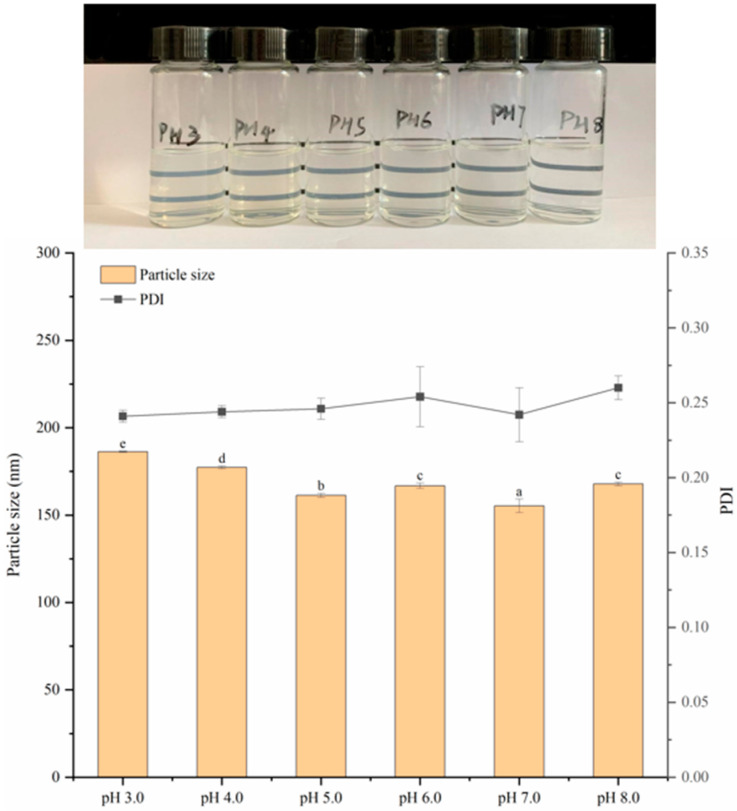
Changes in appearance, particle size and PDI of composite nanoparticles at different pH levels. different lowercase letters denote significant differences (*p* < 0.05).

Different pH levels make the protonation state of some amino acid residues in zein different, resulting in different binding conformations, affecting the charge, structure and interaction of zein and its ligands, which in turn affects the stability of the nanoparticles. The stability of nanoparticles in different pH environments is crucial for the functional activity of allicin. The delivery vector is orally introduced into the gastric fluid (highly acidic) and the small intestine (neutral environment); after a wide range of pH changes, if the nanoparticles do not aggregate, it will be conducive to the effective release of allicin. As shown in Figure 8, with the decrease in pH, the particle size of the nanoparticles increased gradually. At pH 3.0, particle size and PDI reach maximum values of 186.2 nm and 0.241, which may be because in a higher acidic environment, sodium caseinate is coated on the outer layer of nanoparticles and provides a positive charge, and strong electrostatic interaction occurs, resulting in easy aggregation between particles, thereby increasing the average particle size. Normally, particles tend to aggregate and precipitate when the pH is close to the isoelectric point. However, it can be observed in Figure 8 that no obvious aggregation of nanoparticles occurs between 5.0 and 7.0, which may be due to the electrostatic interaction and steric hindrance between sodium caseinate and zein, which makes the nanoparticles stable in different pH environments. The data showed that the stability of zein–Ali–SC was minimally affected by pH, which was similar to that of zein nanoparticles stabilized by other hydrophilic substances [29,30]. The above research results provide theoretical reference for the application of composite nanoparticles in different pH ranges.

#### 3.7.2. Stability Analysis at Different Concentrations of NaCl

**Figure 9:**
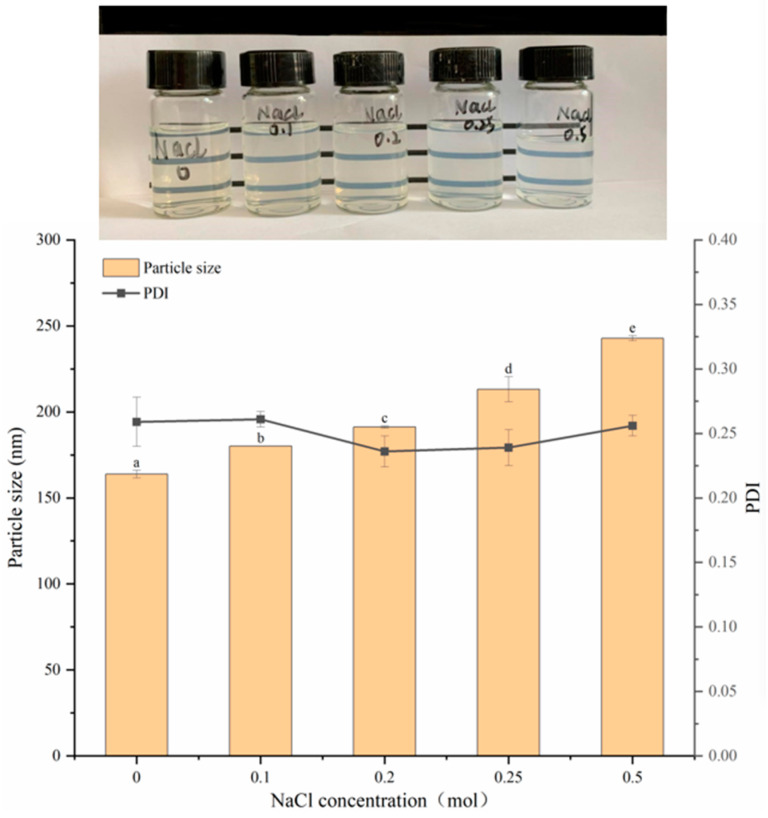
Changes in appearance, particle size and PDI of composite nanoparticles at different concentrations of sodium chloride. different lowercase letters denote significant differences (*p* < 0.05).

Studies have shown that nanoparticles may be affected by the strength of salt ions [31,32], thereby affecting their stability. It can be observed in Figure 9 that the particle size of zein–Ali–SC increases with the increase in NaCl concentration, and the transparency of the dispersion decreases, although there is no obvious precipitation or flocculation. When the concentration of NaCl is 0.5 M, the particle size and PDI are 242.8 nm and 0.256, respectively. This indicates that salt ions exert an electrostatic shielding effect, thereby changing the electrostatic repulsion between particles, thus promoting particle aggregation, and the particle size shows a clear upward trend (*p* < 0.05) [15]. However, the addition of sodium caseinate could slightly increase the colloidal stability of zein particles exposed to NaCl solution, and the dispersion solution did not occur in aggregation [30].

#### 3.7.3. Thermal Stability and Storage Stability Analysis

**Figure 10:**
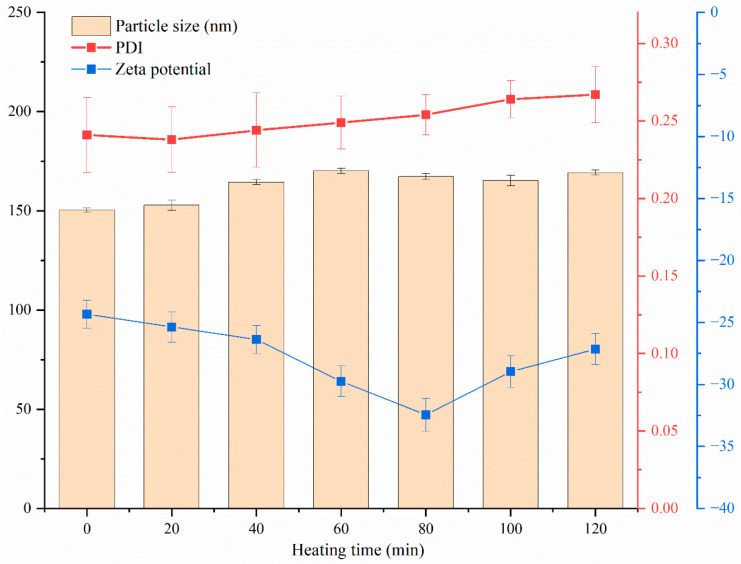
Changes in particle size, PDI and ζ-potential of composite nanoparticles under different heating times.

Heat treatment is a common process in food processing, so it is important to evaluate the thermal stability of nanoparticles. In Figure 10, after heating at 80 °C for different time (20, 40, 60, 80, 100, 120 min), the nanoparticle dispersion solution has a particle size in the range of 150–170 nm, the PDI is less than 0.27, and the ζ-potential is around −25 mV, showing high stability. This may be due to the stable structure formed on the outer layer of zein nanoparticles coated with sodium caseinate. The spatial repulsion and electrostatic repulsion between nanoparticles are conducive to enhancing the protection of active substances during heat treatment by inhibiting the aggregation or decomposition of nanoparticles [11], ensuring the integrity of the nanoparticle structure and not causing the precipitation or degradation of allicin in zein–Ali–SC. Meng et al. showed similar results that curcumin was effectively protected in the hydrophobic cavity of zein and tremella polysaccharide [19].

**Figure 11:**
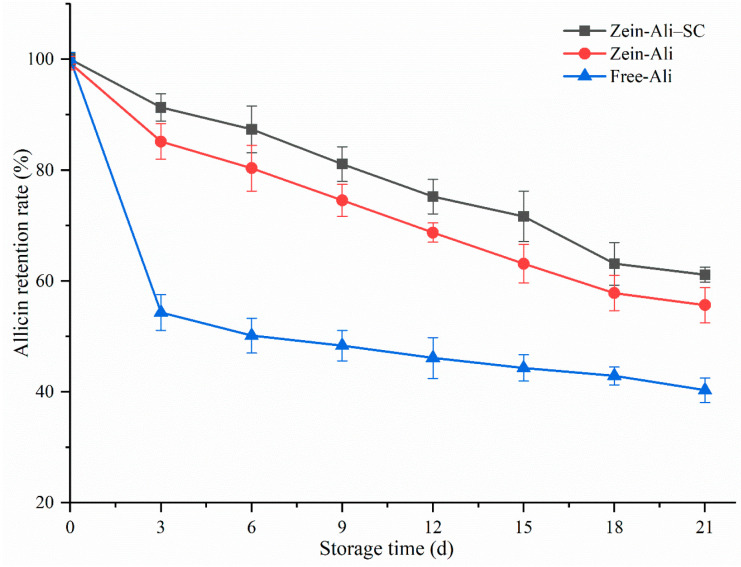
Changes of allicin content under different storage times.

In Figure 11, free allicin began to degrade rapidly on the third day after storage at 4 °C, and the subsequent degradation trend gradually slowed down. When stored on the 21st day, the retention rate dropped to 40.32%; this is similar to the degradation of free allicin obtained by Jiang et al. during the preparation of allicin–whey protein isolate conjugates [33]. However, allicin retention in zein–Ali and zein–Ali–SC decreased slightly, and the retention rates were still high (55.68% and 61.67%) after storage until the 21st day. This may be because allicin enters the water transport part of the composite nanoparticles after being coated with composite nanoparticles, avoiding the degradation of allicin, while the coated sodium caseinate acts as a barrier to shield some of the hydrophobicity of zein nanoparticles, avoiding the aggregation of particles, and further ensuring the stability of allicin. This is similar to the effect of chitosan used by Khan to stabilize zein nanoparticles [34]. This indicates that composite nanoparticles have a good protective effect on allicin, which can effectively encapsulate allicin and improve the stability of allicin.

#### 3.7.4. Analysis of Redispersibility

**Figure 12:**
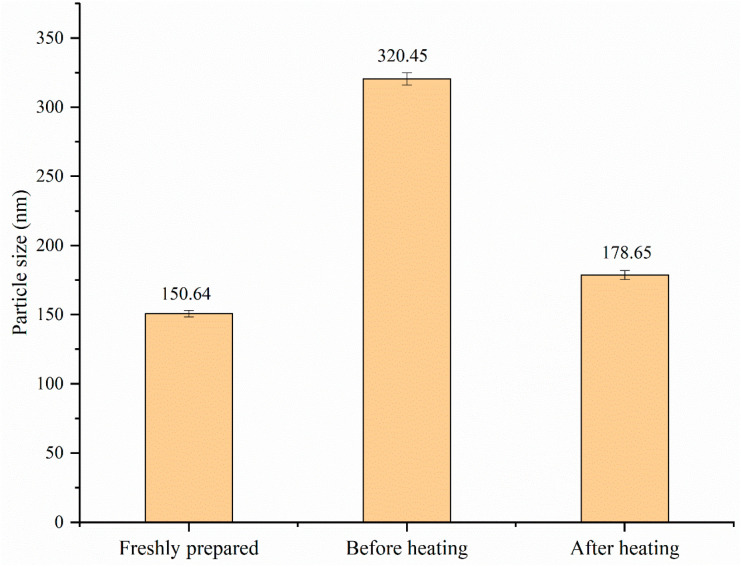
Particle size changes of freshly prepared composite nanoparticle dispersion and freeze-dried sample before and after heating.

For the convenience of transportation, processing, storage, and marketing, freeze-drying zein–Ali–SC into dry-powder products is a feasible and promising method. The redispersibility of dry-powder products is an important characteristic for maintaining good sensory quality of food. Therefore, we evaluated the redispersibility of nanoparticles. From Figure 12, we know that the particle size of freshly prepared nanoparticles is 150.4 nm, and the particle size of the nanoparticle dispersion redissolved in water after freeze-drying treatment is 320.5 nm, which is about twice that of the freshly prepared nanoparticle dispersion. The redispersed nanoparticle dispersion was heated in a water bath at 70 °C for 30 min and then cooled to 25 °C to measure the particle size, which was 178.6 nm. In general, after the freeze-drying treatment, the particle size of the nanoparticles increased due to the agglomeration of the particles. However, after heating, the particle size decreased significantly, resulting in a smaller particle size dispersion, which was similar to the experimental results by Li [35], in which tremella polysaccharide-coated zein–curcumin composite nanoparticles exhibited excellent redispersibility. The results show that the freeze-drying and redispersion processes do not significantly change the particle size of the nanoparticles.

### 3.8. Analysis of Antioxidant Activity

**Figure 13:**
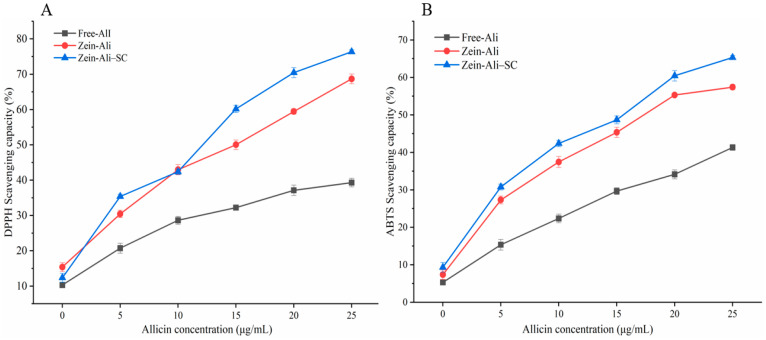
Scavenging activity of DPPH and ABTS free radicals.

Studies have found that allicin has a certain antioxidant activity that can effectively reduce the generation of ROS. In Figure 13A, when allicin concentration is 5 μg/mL, free allicin can clear 20.75% of DPPH, while zein–Ali and zein–Ali–SC can clear 30.45% and 35.39% of DPPH, respectively. When the amount of allicin added increased to 15 μg/mL, the DPPH scavenging ability of zein–Ali (50.04%) and zein–Ali–SC (60.17%) were significantly higher than free allicin (32.23%). When the amount of allicin added increased to 25 μg/mL, the DPPH clearance rate of free allicin was only 39.3%, while the DPPH clearance rate of zein–Ali and zein–Ali–SC were 68.69% and 76.36%, respectively. This indicates that zein–Ali–SC nanoparticles have stronger DPPH scavenging ability than zein–Ali and free allicin while improving the water solubility of allicin, which may be because zein and sodium caseinate-formed nanoparticles have a good synergistic effect on allicin, which is conducive to the antioxidant effect of allicin. At the same time, in order to eliminate the interference of other components in the nanoparticles on the scavenging ability of DPPH radicals, the clearance ability of the composite nanoparticles without allicin was measured. It was found that the scavenging rate of the nanoparticles (10 μg/mL) was only 4.1%, indicating that allicin played a dominant role in scavenging DPPH free radicals.

Similarly, Figure 13B shows that with the increase in allicin content, the clearance ability of zein–Ali and zein–Ali–SC to ABTS gradually increased. When the concentration of allicin was 25 μg/mL, the clearance rates of zein–Ali and zein–Ali–SC to ABTS were 57.42% and 65.32%, respectively, while the highest clearance rate of free allicin was only 41.32%. This indicates that allicin loaded in zein–Ali–SC has a stronger scavenging ability on ABTS free radicals than zein–Ali and free allicin. The coating of nanoparticles can effectively improve the dispersibility of allicin and exhibits better antioxidant capacity.

## 4. Conclusions

In this research, the interaction mode and binding ability between allicin and zein were analyzed by a molecular docking method. The binding energy was −3.7 kcal/mol, and binding sites mainly included PHE178, LEU196 and TYR199. There was hydrophobic interaction between zein and allicin. TYR199 forms π–cation interactions with allicin, which play an important role in stabilizing allicin in zein molecules. Zein–Ali–SC nanoparticles were prepared by antisolvent precipitation and electrostatic deposition. The effect of sodium caseinate addition on the nanoparticles was investigated. The results showed that when W_zein_/W_SC_ = 8:1, the average particle size of the nanoparticles was 151.5 nm, PDI was 0.250, ζ-potential was −35.53 mV, and EE and LC were 86.62% and 7.86%, respectively. The stability of the formed system was the best. SEM showed that the addition of sodium caseinate and allicin had no significant effect on the micromorphology of the composite nanoparticles. The formation mechanism of composite nanoparticles was investigated by fluorescence spectroscopy, FTIR, XRD and TGA: the results indicate that sodium caseinate may be coated on the surface of nanoparticles through electrostatic interactions and form a stable structure. At the same time, the stability of the composite nanoparticles under different conditions was studied: the results show that the particle size was less than 200 nm and the PDI was less than 0.3 under different pH levels, sodium chloride concentrations and heating conditions, showing good stability. When stored at 4 °C for 21 days, the retention rate of allicin was 61.67%, which was 52.9% higher than that of free allicin. After freeze-drying and reheating, the composite nanoparticles had good redispersibility. Antioxidant experiments showed that allicin had stronger clearance ability of DPPH and ABTS free radicals than free allicin. The research results not only contribute to the development of a novel allicin-loaded delivery system, but also provide theoretical support for the application of allicin-loaded nano-delivery systems in food, medicine and other fields.

## Figures and Tables

**Table 1:** Loading characteristics of allicin nanoparticles with different sodium caseinate amounts.

	EE (%)	LC (%)	Particle Size (nm)	PDI	ζ-Potential (mV)
Zein	/	/	121.3 ± 3.8 ^a^	0.220 ± 0.015 ^a^	29.34 ± 1.06 ^d^
Zein–Ali–SC_40:1_	71.54 ± 2.31 ^a^	5.56 ± 0.06 ^a^	132.8 ± 6.5 ^b^	0.222 ± 0.002 ^a^	−30.27 ± 0.61 ^c^
Zein–Ali–SC_20:1_	77.32 ± 3.82 ^b^	5.62 ± 0.02 ^a^	146.0 ± 1.4 ^c^	0.228 ± 0.178 ^a^	−31.79 ± 0.89 ^bc^
Zein–Ali–SC_8:1_	86.62 ± 1.25 ^d^	7.86 ± 0.13 ^c^	151.5 ± 1.1 ^c^	0.250 ± 0.015 ^b^	−35.53 ± 1.06 ^a^
Zein–Ali–SC_4:1_	85.17 ± 2.49 ^d^	6.75 ± 0.25 ^b^	176.6 ± 0.5 ^d^	0.272 ± 0.012 ^c^	−33.18 ± 0.66 ^b^
Zein–Ali–SC_2:1_	81.38 ± 2.12 ^c^	6.84 ± 0.05 ^b^	196.5 ± 2.5 ^e^	0.291 ± 0.010 ^d^	−32.65 ± 1.57 ^b^

Note: Different lowercase letters in the table indicate significant differences (*p* < 0.05).

## Data Availability

The original contributions presented in the study are included in the article, further inquiries can be directed to the corresponding author.
